# Thermal Effects of Burr and Saw Cutting Tools in Total Knee Arthroplasty

**DOI:** 10.1155/aort/4182322

**Published:** 2026-08-17

**Authors:** Vivek Sharma, Mahbub Alam, Dhiraj Sharma, Lokesh Sharoff, Barbara Pierscionek, Richard Smith, Timothy Parratt

**Affiliations:** ^1^ Department of Trauma and Orthopaedic Surgery, East Suffolk and North Essex NHS Foundation Trust Hospital, Colchester, UK; ^2^ Department of Trauma and Orthopaedic Surgery, Imperial College Healthcare NHS Trust, London, UK, nhs.uk; ^3^ Faculty of Health, Medicine and Social Care, Anglia Ruskin University, Chelmsford, UK, anglia.ac.uk; ^4^ Department of Nephrology, East Suffolk and North Essex NHS Foundation Trust Hospital, Colchester, UK

## Abstract

**Aims:**

Aseptic loosening after total knee arthroplasty (TKA) can follow thermal bone injury produced by power tools. This study compared the intraoperative bone temperatures generated by rotary burrs used in robotic‐assisted TKA (rTKA) with those from oscillating saws used in conventional TKA (cTKA).

**Methods:**

A single‐centre observational trial employed infrared thermography (30 Hz, ±0.05°C) to record surface temperatures during the distal femoral cut in 14 primary TKAs (rTKA = 6, cTKA = 8). Burr cuts used a 6‐mm spherical burr at 80,000 rpm; saw cuts used Stryker sagittal blades. Peak and mean temperatures and the time spent above 47°C, 56°C and 70°C were extracted from the recordings and compared with Mann–Whitney tests.

**Results:**

All procedures exceeded the osteonecrosis threshold (47°C); maximal temperatures surpassed 90°C in every burr case and in 5/8 saw cases. Median cutting time was 241 s with the burr versus 26 s with the saw (*p* = 0.002). The bone remained above 47°C for a median 107 s (IQR 138) with the burr, nearly four‐fold longer than with the saw (8 s; IQR 10; *p* < 0.001). Similar differences were observed above 56°C (65 s vs. 3 s) and 70°C (15 s vs. 0.5 s). Mean temperatures (∼51°C) did not differ significantly between devices.

**Conclusion:**

Rotary burrs and oscillating saws both generate potentially harmful heat during TKA, but burrs markedly prolong exposure to cytotoxic temperatures and lengthen operative time. These findings suggest a higher theoretical risk of thermally mediated bone injury and subsequent implant loosening when burrs are used without active cooling. Larger controlled studies should assess mitigation strategies such as irrigation, cooled instrumentation or modified cutting protocols.

## 1. Introduction

Total knee arthroplasty (TKA) involves removing the degenerative articular surface of the knee joint and reconstructing it with prosthetic materials. TKA is the gold standard of care for patients with end‐stage osteoarthritis who are refractory to conservative treatment. It is one of the most successful surgical interventions, with an 85% implant survivorship at 15–25 years. Patient‐reported satisfaction rates range from 80% to 100%, determined primarily by postoperative functional outcomes and pain relief [1, 2].

TKA procedures are on the rise in developed nations, driven by an ageing population and increasing obesity rates, which result in higher rates of osteoarthritis [3]. The United States projects nearly 3.5 million procedures annually by 2030, while the United Kingdom projects 1.2 million [4, 5]. Compounding this, TKAs are being performed in increasingly younger patients. Traditionally reserved for the elderly, there is now growing demand for improved quality of life, leading to TKRs being performed on patients under 65, who now represent 40% of cases [6]. These younger patients have higher physical demands and longer life expectancies, leading to a more than twice higher revision rate (52% vs. 29%) [7].

The most common cause of TKA revision is aseptic loosening, caused by the laxation of the femoral or tibial components from the bone in the absence of infection [8, 9]. Thermal injury to bone is time‐ and temperature‐dependent; less than 44°C causes no thermal injury, but temperatures above 47°C for 60 s are associated with osteonecrosis [10]. Brief thermal spikes can induce osteonecrosis: 30 s at 50°C damages bone up to 1 mm from the heat source, while > 70°C causes immediate cell death [11]. Alkaline phosphatase (ALP), an enzyme crucial for biomineralization, begins to denature at 56°C [12]. Aseptic loosening occurs following necrosis, where reparative fibrous and granulation tissue forms around the implant, developing into a dense fibrous pseudomembrane [13]. Autopsy studies indicate that well‐fixed, stable implants usually have little fibrous tissue between the implant and the surrounding bone, whereas loose implants are encased in a thick fibrous pseudomembrane [14].

Elevated temperatures during bone cutting procedures may contribute to bone implant surface resorption, ultimately leading to prosthetic loosening. Baker et al. recorded temperatures as high as 89°C during femoral head preparation for hip resurfacing procedures, with more than 47°C recorded in 28 of the 35 patients in the study [15]. Similarly, McCann et al. found temperatures exceeding the physiological threshold for osteonecrosis during humeral reaming for total shoulder resurfacing [16].

In TKA, oscillating saws have been the primary cutting modality for most surgeons. However, the rise of robotic orthopaedic‐assisted TKA systems has increased the prevalence of burr cutting tools due to their greater control and accuracy [17]. There is a paucity of evidence about the use of burrs in orthopaedic surgery, with most literature arising from the dental field.

Tawy et al.’s bovine femora study showed burrs maintained temperatures above 47°C for > 60 s despite irrigation, while saws cooled effectively below this threshold [18]. However, in vitro models inadequately represent surgical conditions, particularly the sclerotic bone of arthritic joints. Living bone studies are essential as blood flow, synovial fluid, and surrounding tissues enhance heat dissipation.

No studies have investigated the maximum temperature generated by sawing and burring during in human in vivo TKA procedures, where the presence of synovial tissue and blood in living bone may provide a similar cooling effect to irrigation. To enhance our understanding of the relationship between orthopaedic power tools and bone temperature during TKA bone preparation in vivo, our objectives are twofold: Firstly, to establish the temperatures attained during sawing and burring, and secondly, to compare temperature differences between in vivo sawing and burring during bone preparation. We hypothesised that there were no differences in peak temperatures generated by burr and saw devices.

## 2. Methodology

### 2.1. Ethical Approval and Study Design

The study protocol was submitted via UK Integrated Research Application System (IRAS‐334601) and approved by the East of England Research Ethics Committee (24/PR/0381) in April 2024. This was a single‐centre, prospective, observational study. All reporting follows the Strengthening the Reporting of Observational Studies in Epidemiology (STROBE) guidelines; a completed STROBE checklist is provided as Supporting Information (available here).

### 2.2. Trial Design and Participant Recruitment

This single‐centre observational clinical research trial involved patients scheduled for TKA due to osteoarthritis. Eligible participants were over 18 years with symptomatic osteoarthritis, considered for robotic assisted (rTKA—using burr as a cutting device) or conventional TKA (cTKA—using saw as a cutting device). Exclusion criteria included distorted bone anatomy, previous knee surgery, and bone health–affecting diseases. Participants received standard care. Assignment to rTKA or cTKA was determined by logistical factors: rTKA was performed only when both a surgeon credentialled in the robotic‐assisted technique and the requisite theatre staff and equipment (CORI surgical system, Smith and Nephew) were simultaneously available. When these conditions were not met, patients underwent cTKA. This allocation was not randomised. The following demographic data were collected during clinic appointments: age, comorbidities, gender, and presenting complaint. Additionally, the operative notes were reviewed to assess any adverse events or the use of water irrigation during the surgery. All procedures were performed under a tourniquet.

**FIGURE 1:**
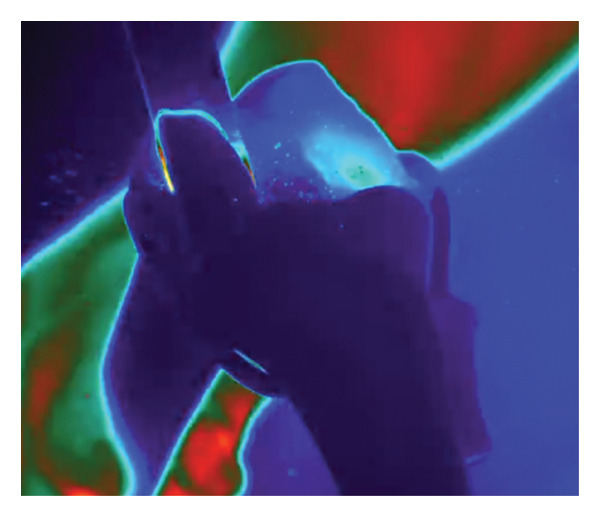
Image captures were taken using the Thermovision A320 camera during simulation surgeries with dry bone cutting. For the temperature analysis, the cutting tool was excluded from the measurements.

In July 2024, all eligible patients who consented were recruited for the study. Informed consent encompassed permission for infrared thermography and image recording during their TKA procedures. To ensure consistency and accuracy, all procedures were conducted by a consultant orthopaedic surgeon using standard surgical techniques (Figure 1).

### 2.3. Infrared Thermography and Surgical Procedure

A thermal imaging camera (Thermovision A320, Flir Systems Inc., Wilsonville, Oregon, USA) was placed 2 m from the operative field to record surface temperatures in real time with an accuracy of ±0.05°C. The camera was calibrated to ambient room temperature and humidity before use. It was connected to a laptop running ThermaCAM Researcher Pro software (Version 2.8).

The distal femur was selected for analysis due to consistent surgical exposure and the ability to obtain unobstructed thermographic measurements. Tibial resections were not included due to challenges in maintaining a consistent field of view and avoiding obstruction during instrumentation. No irrigation was used whilst performing the resections as this was not standard practice.

The Thermovision A320 camera was placed 2 m outside the sterile field and focused on the operative site. Recording commenced upon exposure of the distal femur and continued until after the distal femoral cut was complete. Theatre spotlights were turned off during femoral preparation to reduce thermal artefact. For cTKA, Stryker Performance SeriesTM 81 sagittal blades (cut edge: 25.0 mm, cut depth: 90.0 mm, thickness: 0.97 mm) were used. For rTKA, a CORI surgical system handheld robotic device (Smith and Nephew) with 6‐mm‐diameter spherical burrs was used at 80,000 rpm. New burrs and saw blades were used for each procedure.

### 2.4. Data Collection and Analysis

Thermal imaging data were recorded at a frequency of 30 Hz, and the maximum bone temperature was recorded for each image. Before each use, a single camera calibrated to detect temperatures with an accuracy of 0.05 ^o^C between 10 and 90^o^C was used to capture all measurements throughout the study. The calibration process accounted for ambient room temperature and humidity, adhering to the manufacturer’s specified instructions. Human bone emissivity was assumed to be 0.99, and the bone surface area was the manually defined region of interest, excluding the cutting tool [19]. This step calibrates the equipment and allows accurate and reproducible temperature measurement. Data were exported to a CSV file and analysed using Python 3.0 in a Jupyter Notebook. As per ethical approval, all imaging data and CSV files were stored on password‐protected NHS computer. A power calculation, based on cadaveric data, estimated a required sample size of at least 9 patients per group to achieve a power of 0.8 with a significance level of 0.05 to detect a 2°C difference between cutting devices. The significance due to cutting instrument type was obtained using the Mann–Whitney test due to the small sample size.

## 3. Results

**FIGURE 2:**
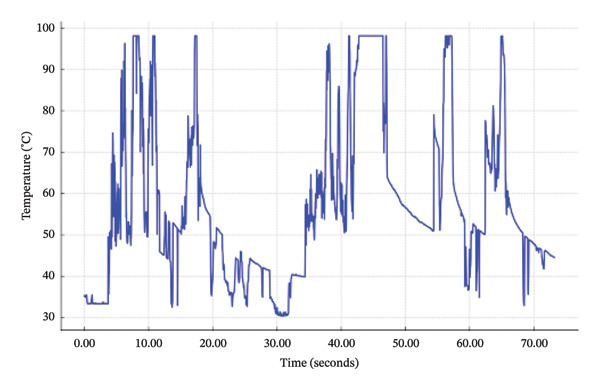
Time series graph showing the extrapolated maximum temperatures recorded while a patient underwent the distal cut of the femur using a saw device.

**FIGURE 3:**
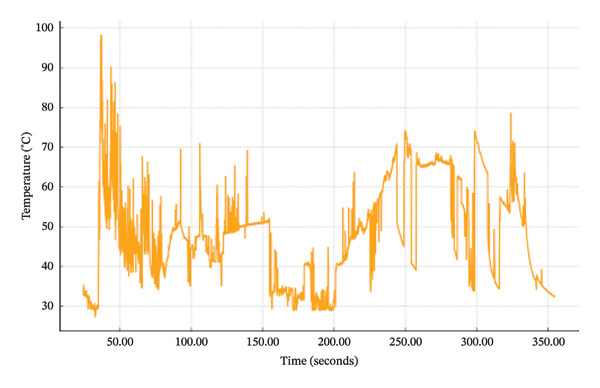
Time series graph showing the extrapolated maximum temperatures recorded while a patient underwent the distal cut of the femur using a burr device.

In July 2024, 15 patients were recruited from the Orthopaedic Outpatients Clinic. One case was excluded from the analysis due to the camera being obstructed. Of the remaining patients, 6 received rTKA, where a burr (3 male, 60%) was used as the cutting device, and 8 received a cTKA (4 male, 50%), where a saw was used. The median age was 76 in the rTKA group and 69 in the cTKA group. The raw data from each recording were extrapolated into time series data, as shown in Figures 2 and 3. No adverse events were reported during surgery or the patients’ inpatient stays.

### 3.1. Baseline Characteristics

The mean temperatures generated were not significantly associated with gender (*p* = 0.41), age (*r* = 0.25, *p* = 0.38), or BMI (*r* = −0.59, *p* = 0.056). Additionally, there were no significant differences between the burr and saw groups regarding age (*p* = 0.65), gender (chi‐square test, *p* = 1.00) or BMI (*p* = 0.078).

### 3.2. Time to Complete Distal Cut

The median time to complete the distal cut was significantly longer in the burr group compared to the saw group. In the saw group, the median time was 25.76 s (IQR = 28.79, range = 46.75), whereas in the burr group, it was 241 s (IQR = 154, range = 253). This difference was statistically significant (*p* = 0.0016).

### 3.3. Mean and Maximum Temperatures

The median temperature in the saw group was 50.52°C (IQR = 6.81, range = 18.15), while in the burr group, it was 51.11°C (IQR = 2.49, range = 5.62). There was no significant difference in median temperatures between the saw and burr groups (*p* = 0.95).

Maximum temperatures exceeding 90°C were recorded in 5 out of 8 patients in the saw group, with the remaining 2 patients recording maximum temperatures between 69°C and 84°C. In the burr group, all 6 patients recorded temperatures exceeding 90°C.

### 3.4. Time Above Threshold Temperatures

The median times above 47°C and 56°C in the saw group were 8.06 s (IQR = 9.82, range = 20.76) and 3.4 s (IQR = 5.30, range = 8.16), respectively. In the burr group, the median distal cut times were 106.88 s (IQR = 138.14, range = 179.64) and 64.52 s (IQR = 107.77, range = 146.96), respectively. The time spent above the 47°C and 56°C thresholds was significantly longer in the burr group compared to the saw group (both *p* = 0.00067).

For temperatures exceeding 70°C, the median time was 0.46 s (IQR = 2.31, range = 5.36) in the saw group and 15.0 s (IQR = 20.76, range = 50.88) in the burr group. This difference was also statistically significant (*p* = 0.013).

## 4. Discussion

The aim of this study was to evaluate the thermal impact of burr and saw power cutting tools during the distal cut in TKA. Our findings revealed that intraoperative temperatures frequently exceeded the necrosis threshold of 47°C, with maximum temperatures surpassing 90°C in most procedures. Mean temperatures were not significantly higher in the burr cutting group, in support of our initial hypothesis.

Upon initiating bone cutting, both burr and saw devices caused a sudden rise in bone temperatures due to the frictional forces at the bone–tool interface, as illustrated in Figures 2 and 3. However, there was no significant difference in the temperatures generated by the two cutting devices. This is consistent with the findings of Tawy et al., who reported that both burr and saw devices produced mean temperatures around 50°C without irrigation. These results suggest that the presence of synovial fluid and blood in living cancellous bone does not provide the cooling effect previously proposed by authors such as Tawy et al. and Sugita et al. [18, 20].

Although the mean temperatures did not differ significantly, the temporal temperatures generated during the bone cutting process did. The saw device caused sudden spikes in temperature as it cut through the denser cortical bone, which has a higher mineral content and thermal conductivity, before dropping as it moved into the less dense cancellous bone [21]. In contrast, the burr maintained high temperatures throughout the distal cut, gradually cutting through a larger surface area of the cortical bone, leading to prolonged exposure to elevated temperatures. This is reflected in the duration of time the bone spent above the threshold for necrosis; the burr group remained above the 47°C threshold nearly four times longer than the saw group, and around eight times longer for the 56°C threshold [22].

There is no formally accepted threshold for osteonecrosis, but Eriksson and Albrektsson demonstrated that it could occur at temperatures as low as 47°C in a rabbit model, while Lundskog et al. found the threshold to be 50°C for 30 s [23, 24]. It is likely that the threshold lies between 47°C and 55°C for 1 min [25]. However, these studies were primarily conducted in animal models, so the exact threshold for human bone necrosis remains unclear. ALP begins to denature at 56°C but only after sustained exposure for 15 min.

It should be emphasised that our study only assessed the distal cut, and additional thermal insult may be incurred from the additional femoral cuts, albeit in different direct contact areas. The implications for bone necrosis are uncertain, as it is unlikely that any specific section of the bone surface would be exposed to such high temperatures for a sustained consecutive.

Nevertheless, the thermal insult from power tools during surgery can compound the heat generated by the cement used to anchor TKA prostheses. In the United Kingdom, over 83% of primary TKA procedures use cement for prosthesis fixation [26]. The polymerisation of polymethylmethacrylate (PMMA) bone cement during the curing process is an exothermic reaction that releases heat. Kurata et al. used a three‐dimensional finite element model to predict a maximum temperature rise of 50°C at the cement–tissue interface, which is sufficient to cause thermal damage to osteocytes [27]. This additional heat from PMMA may exacerbate the thermal insult already caused by power tools.

Thermal osteonecrosis and the subsequent remodelling of the surrounding bone can result in the loss of microlock at the cement–bone interface, potentially leading to prosthesis loosening [15]. This issue is even more critical for cementless TKA implants, which rely on the migration of osteoblasts and mesenchymal cells for osseointegration with the roughened surface of the implant. Cementless implants with “biological” fixation offer the potential for durable and stable fixation, reducing the risk of aseptic loosening [28]. However, the high temperatures generated by power tools remain a significant concern that must be properly addressed.

The median cutting time for the distal femur was almost seven times longer when using a burr compared to a saw. Although only the distal cut was measured, extrapolating these times to include the four additional femoral cuts and the tibial cut would significantly increase the overall operative time. This increase in operative time is supported by Tompkins et al., who conducted a retrospective cohort study of 62,221 patients and found that rTKA procedures took longer than cTKA, with in‐room/out‐of‐room times averaging 32 min longer and procedure times being 8 min longer on average [29]. Increased operative time has financial implications, costing approximately $2800.00 per hour of theatre time, and may reduce the number of cases that can be performed in a day, leading to even greater financial consequences [30]. Additionally, longer surgical times also mean extended anaesthesia duration, which increases the risk of perioperative complications [31].

### 4.1. Mitigation Strategies: Areas for Future Research

Several strategies warrant evaluation as areas for future research rather than established solutions: intraoperative room temperature water irrigation, which may reduce peak temperatures but had limited effect on burrs in vitro [18]; cooled instrumentation, including integrated irrigant delivery and modified cutting parameters such as rotational speed, intermittent cutting cycles or altered burr geometry. None of these is currently supported by evidence sufficient to recommend routine clinical adoption, and each should be prioritised in adequately powered controlled studies with direct biological and implant‐survivorship outcomes.

This study was small and exploratory in nature. The sample size did not meet the predefined power calculation target, which limits the statistical power and restricts the conclusions that can be drawn. Although our study recorded temperatures higher than previously defined osteonecrosis thresholds, we were unable to provide direct evidence of bone necrosis as no histopathological examination of distal cut debris was performed. The absence of histopathological correlation limits the ability to directly link observed temperatures to biological bone injury. However, recent histopathological data from Londhe et al. provide useful contextual support, demonstrating no significant difference in osteocyte viability or depth of thermal injury between oscillating saw and burr techniques at the tibial cut surface. Whilst this offers important biological correlation to thermal effects observed during bone cutting, it should be noted that their study assessed a different anatomical site and methodology [32].

Another limitation is that the infrared camera was only able to capture temperature data when the saw blade or burr was in view, which occurred when cutting the cortical surface. This limitation contributed to the study being restricted to distal femoral cuts**.** Thermal effects during tibial preparation, which are highly relevant to implant fixation and aseptic loosening, were not assessed. This limits the generalisability of our findings, particularly in the context of tibial component failure. Future studies should aim to include both femoral and tibial resections to provide a more comprehensive evaluation of intraoperative thermal effects.

We were also unable to measure the temperature and effect on the bone below the surface. Previous studies have used thermocouples placed into drill holes near the cutting surface or within drill bits or saw blades to estimate surface temperatures [33]. However, these methods have significant limitations due to the low thermal conductivity and diffusivity of the bone, which creates a steep temperature gradient around the cutting site [34]. As a result, such studies may have underestimated temperatures due to factors such as the minimum distance from the cutting site, the size of the thermocouple, thermal contact resistance and potential errors in positioning the thermocouple. Additionally, drilling extra bone holes in clinical practice would not be ethically acceptable.

This was not a controlled study. Allocation to rTKA or cTKA was based entirely on the logistical availability of a trained robotic surgeon and equipment, not on any clinical or patient‐level criterion. This nonrandom allocation introduces selection bias and means that differences between groups beyond the cutting modality cannot be fully excluded. Additional uncontrolled variables include surgeon technique and force applied to the cutting device, as well as bone mineral density and degree of subchondral sclerosis, all of which may affect heat generation independently of the cutting modality.

## 5. Conclusions

This small, exploratory observational study provides preliminary evidence that both burr and saw devices may generate temperatures exceeding thresholds associated with thermal bone injury, with burrs demonstrating markedly prolonged exposure. The small sample size, observational design, nonrandom allocation and absence of direct histopathological evidence all limit the conclusions that can be drawn. These findings should be interpreted cautiously and regarded as hypothesis‐generating. Future larger controlled studies should address these limitations and investigate potential mitigation strategies including intraoperative irrigation, cooled instrumentation and modified cutting parameters.

## Funding

No funding was received.

## Ethics Statement

The study was registered prospectively with ClinicalTrials.gov and approved by the East of England Research Ethics Committee (24/PR/0381) in April 2024.

## Conflicts of Interest

The authors declare no conflicts of interest.

## Supporting Information

Additional supporting information can be found online in the Supporting Information section.

## Supporting information


**Supporting Information** The Strengthening the Reporting of Observational Studies in Epidemiology (STROBE) Statement checklist was used to evaluate the reporting quality of this observational study.

## Data Availability

The data that support the findings of this study are available on request from the corresponding author. The data are not publicly available due to privacy or ethical restrictions.
