# Supplementary material for: Thermal Effects of Burr and Saw Cutting Tools in Total Knee Arthroplasty
Source: Adv Orthop. 2026 Aug 17;2026:4182322. doi: 10.1155/aort/4182322 (PMC13481704; doi:10.1155/aort/4182322)
Supplement: Supplementary file 1 [file AORT-2026-4182322-s001.docx]

**Supplementary Material**

**STROBE Statement — Checklist of items that should be included in reports of observational studies**

*Study design: Observational cohort study (cross-sectional intraoperative analysis)*

| **Section/Topic** | **Item** | **Recommendation** | **Reported on page/location** |
| --- | --- | --- | --- |
| Title and abstract | 1 | (a) Indicate the study's design with a commonly used term in the title or the abstract. (b) Provide in the abstract an informative and balanced summary of what was done and what was found. | Title; Abstract – Methods paragraph. |
| Introduction: Background/rationale | 2 | Explain the scientific background and rationale for the investigation being reported. | Introduction, paragraphs 1–5. |
| Introduction: Objectives | 3 | State specific objectives, including any pre-specified hypotheses. | Introduction, final paragraph. |
| Methods: Study design | 4 | Present key elements of study design at the beginning of the methods section. | Methods – Trial Design and Participant Recruitment, paragraph 1. |
| Methods: Setting | 5 | Describe the setting, locations, and relevant dates, including periods of recruitment, exposure, follow-up, and data collection. | Methods – Trial Design and Participant Recruitment. |
| Methods: Participants | 6 | (a) Give the eligibility criteria, and the sources and methods of selection of participants. (b) Describe methods of follow-up. | Methods – Trial Design and Participant Recruitment. |
| Methods: Variables | 7 | Clearly define all outcomes, exposures, predictors, potential confounders, and effect modifiers. Give diagnostic criteria if applicable. | Methods – Infrared Thermography; Data Collection and Analysis. |
| Methods: Data sources / measurement | 8 | For each variable of interest, give sources of data and details of methods of assessment. | Methods – Infrared Thermography and Surgical Procedure. |
| Methods: Bias | 9 | Describe any efforts to address potential sources of bias. | Discussion – Limitations. |
| Methods: Study size | 10 | Explain how the study size was arrived at. | Methods – Data Collection and Analysis (power calculation). |
| Methods: Quantitative variables | 11 | Explain how quantitative variables were handled in the analyses. | Methods – Data Collection and Analysis. |
| Methods: Statistical methods | 12 | (a) Describe all statistical methods, including those used to control for confounding. (b) Describe any methods used to examine subgroups and interactions. (c) Explain how missing data were addressed. (d) If applicable, describe methods used to address loss to follow-up. (e) Describe any sensitivity analyses. | Methods – Data Collection and Analysis. |
| Results: Participants | 13 | (a) Report numbers of individuals at each stage of study. (b) Give reasons for non-participation at each stage. (c) Consider use of a flow diagram. | Results, paragraph 1. |
| Results: Descriptive data | 14 | (a) Give characteristics of study participants and information on exposures and potential confounders. (b) Indicate number of participants with missing data for each variable. | Results – Baseline characteristics. |
| Results: Outcome data | 15 | Report numbers of outcome events or summary measures. | Results – Time above threshold temperatures; Figures 2 and 3. |
| Results: Main results | 16 | (a) Give unadjusted estimates and, if applicable, confounder-adjusted estimates. (b) Report category boundaries when continuous variables were categorised. (c) If relevant, consider translating estimates of relative risk into absolute risk for a meaningful time period. | Results – Mean and maximum temperatures; Time above threshold temperatures. |
| Results: Other analyses | 17 | Report other analyses done — e.g., analyses of subgroups and interactions, and sensitivity analyses. | Results – Baseline characteristics. |
| Discussion: Key results | 18 | Summarise key results with reference to study objectives. | Discussion, paragraph 1. |
| Discussion: Limitations | 19 | Discuss limitations of the study, taking into account sources of potential bias or imprecision. Discuss both direction and magnitude of any potential bias. | Discussion – Limitations. |
| Discussion: Interpretation | 20 | Give a cautious overall interpretation of results considering objectives, limitations, multiplicity of analyses, results from similar studies, and other relevant evidence. | Discussion. |
| Discussion: Generalisability | 21 | Discuss the generalisability (external validity) of the study results. | Discussion – Limitations. |
| Other information: Funding | 22 | Give the source of funding and the role of the funders for the present study and, if applicable, for the original study on which the present article is based. | Funding statement. |

*Von Elm E, Altman DG, Egger M, Pocock SJ, Gøtzsche PC, Vandenbroucke JP, for the STROBE Initiative. The Strengthening the Reporting of Observational Studies in Epidemiology (STROBE) Statement: Guidelines for Reporting Observational Studies. Ann Intern Med. 2007;147:573–577.*
